# Neuroactive Multifunctional Tacrine Congeners with Cholinesterase, Anti-Amyloid Aggregation and Neuroprotective Properties

**DOI:** 10.3390/ph4020382

**Published:** 2011-02-18

**Authors:** Maria Kozurkova, Slavka Hamulakova, Zuzana Gazova, Helena Paulikova, Pavol Kristian

**Affiliations:** 1 Department of Biochemistry, Institute of Chemistry, P. J. Šafárik University, Moyzesova 11, Košice, Slovakia; 2 Department of Organic Chemistry, Institute of Chemistry, P. J. Šafárik University, Moyzesova 11, Košice, Slovakia; E-Mails: slavka.hamulakova@upjs.sk (S.H.); pavol.kristian@upjs.sk (P.K.); 3 Department of Biophysics, Institute of Experimental Physics, Slovak Academy of Sciences, Watsonova 47, Košice, Slovakia; E-Mail: gazova@saske.sk (Z.G.); 4 Department of Biochemistry and Microbiology, Faculty of Chemical and Food Technology, Slovak University of Technology, Radlinského 9, Bratislava, Slovakia; E-Mail: helena.paulikova@stuba.sk (H.P.)

**Keywords:** tacrine, acetylcholinesterase inhibitor, amyloid aggregation, Alzheimer's disease

## Abstract

The review summarizes research into the highly relevant topics of cholinesterase and amyloid aggregation inhibitors connected to tacrine congeners, both of which are associated with neurogenerative diseases. Various opinions will be discussed regarding the dual binding site inhibitors which are characterized by increased inhibitor potency against acetylcholin/butyrylcholine esterase and amyloid formation. It is suggested that these compounds can both raise levels of acetylcholine by binding to the active site, and also prevent amyloid aggregation. In connection with this problem, the mono/dual binding of the multifunctional derivatives of tacrine, their mode of action and their neuroprotective activities are reported. The influence of low molecular compounds on protein amyloid aggregation, which might be considered as a potential therapeutic strategy in the treatment of Alzheimer's disease is also reported. Finally, attention is paid to some physico-chemical factors, such as desolvation energies describing the transfer of the substrate solvated by water, the metal-chelating properties of biometals reacting with amyloid precursor protein, amyloid beta peptide and tau protein.

## 1. Introduction

Tacrine was first described pharmacologically by Shaw and Bently in Australia [1] in 1949 as an analeptic capable of causing rapid arousal in morphinized dogs and cats. Early clinical applications of tacrine were in the treatment of anesthetic induced delirium [2] and the potentiation of the muscle relaxing effects of succinylcholine [3].

Heilbronn in 1961 [4] was the first to describe the effect of tacrine upon acetylcholinesterase (AChE) and butyrylcholinesterase (BuChE). She demonstrated that the compounds act as a reversible inhibitor being partly competitive with the substrate acetylcholine (ACh) and a more potent inhibitor of BuChE than of AChE [5].

Other than being an enzyme inhibitor, tacrine was found to have other mechanisms which could explain its cholinergic effect. In electrophysiological studies with neurons of *Lymnaea stagnalis*, tacrine inhibits the slow outward K^+^ current and consequently increases the duration of the action potentials. Moreover, tacrine inhibits the uptake of noradrenaline, dopamine and serotonine. Such uptake inhibition does not seem to occur at the level of the axonal membrane, but instead at the level of the monoaminergic storage granules [6], and thus tacrine might well have some monoaminergic effect [7]. Tacrine was one of the first ChEI to be tested clinically for the treatment of Alzheimer's disease (AD). Numerous studies [8] had been performed on a small group of patients, with physostigmin alone or in combination with lecithin. The initial report by Summers *et al*. [9] which suggests that a combined oral treatment of tacrine and lecithin improved the condition of patients with AD has attracted considerable interest, although later studies have failed to demonstrate such marked improvements. Several reasons have been proposed to explain the negative results of these studies; the small sample size used in the research being among the most significant, and the fact that many of the subjects chosen for the study were already in advanced stages of the disease. Also, multiple transmitter deficits are probably present in AD in addition to a loss of neurons in critical areas of the brain, and counteracting the former with tacrine alone does not bring about clinically demonstrable benefits [7]. There is also a high incidence of side effects in the use of tacrine–autonomic symptoms such as gastrointestinal disturbance and hypotension. Additionally, the therapeutic index of tacrine is so small that it is both critical and difficult to find the optimum dose, as individual patients vary greatly in their responsiveness to the treatment [10-12].

In this review, we will summarize the research into the development of tacrine derivatives with cholinesterase and amyloid aggregation inhibiting activity in addition to discussing their neuroprotective properties. Although many reviews have been published on this topic with medicinal or pharmaceutical focuses, we will try to contribute from a different view of point, namely from a bioorganic perspective.

## 2. Cholinesterse Inhibitors

Acetylcholinesterase (AChE, E.C.3.1.1.7) and butyrylcholinesterase (BuChE, E.C.3.1.1.8) are enzymes which constitute the group of the cholinesterases. Acetylcholinesterase hydrolyses acetylcholine (AChE) and is mainly associated with nerves and muscles, being typically found on the synapsies, while butyrylcholinesterase, also known as plasma cholinesterase or pseudocholinesterase, hydrolyses butyrylcholine and is synthesized by the liver, being found in large concentrations in serum [13].

All current kinetic models for AChE propose the existence of at least two substrate-binding sites; the active site, near the bottom of the active-site gorge, and the peripheral anionic site (PAS), near its entrance. Binding the ligands at the PAS affects catalytic activity [14-15]. The active site is composed of two subsites. In the catalytic anionic subsite (CAS), it has been proposed that the choline moiety of AChE is stabilized principally via a cation-π interaction with Trp84, and also interacts with Glu199 and Phe330 [16]. A similar cation-π interaction occurs in human BuChE, where Trp82 interacts with the product choline and the substrate butyrylthiocholine [17]. The PAS contain three principal amino acids; Trp279, Tyr70 and Asp72. This was established by site-directed mutagenesis and by binding of inhibitors. AChE, one of the most crucial enzymes for nerve response and function, catalyzes the hydrolysis of acylcholinesters with a relative specificity for acetylcholine.

Szegletes *et al*. [18] demonstrated that the primary physiologic role of the PAS is to accelerate the hydrolysis of acetylcholine at low concentrations of acetylcholine. The gorge of the active site is so deep and broad that it can bind many different substrates and inhibitors. The initial step in the catalytic pathway is substrate binding to the PAS, therefore the designing of new type inhibitors of AChE should be based on the hypothesis of dual binding of AChE inhibitors. The PAS can act as an uncompetitive inhibitor binding site. Strong binding of inhibitors at the peripheral site may hinder the entry of acetylcholine to the enzyme gorge. Such types of inhibitors which are able to bind to PAS can act as a steric blocker.

The hydrolysis reaction proceeds with the nucleophilic attack on the carbonyl carbon, acylating the enzyme and liberating choline. This is followed by a rapid hydrolysis of the acylated enzyme yielding acetic acid, and the restoration of the esteratic site. The pathway is similar to a pipeline, where the substrate goes in one end and the products come out at the other end through conformational changes, along with the effect of hydrophobic and electrostatic forces. In mammals, the alternative splicing of a single gene gives rise to three different variants of AChE (types R, H/E, and T/S), which possess the same catalytic activity, but with different tissue specific distributions. AChE is a glycoprotein which is present in cells as either a monomer or an oligomer, depending on the type of cells and on the variant of enzyme, but all of them is membrane-bound.

Recent findings indicate that AChE is involved not only in the correct functioning of the central and peripheral nervous systems, but that it is a ubiquitous molecule which takes part in the regulation of several other processes such as cell growth, locomotion or apoptosis [19].

While AChE has a well established “classical” esterase activity, the physiologic role of BuChE is still controversial. BuChE is an enzyme which is also involved in cholinergic neurotransmission, and which has received increasing attention in recent years [20].

However, BuChE may have a compensatory role in the hydrolysis of ACh in brains with degenerative changes. Although BuChE activity is lower than that of AChE in the normal human brain, BuChE activity is greatly increased in AD [21-22]. It is possible that these divergent changes are related to the loss of cholinergic synapses and neurons and the increases in glial cells in the AD brain, which suggests that inhibition of BuChE (in adition to AChE) may become more important as AD progresses. This has raised the hypothesis that inhibitory action on both enzymes could lead to improved therapeutic benefits [20,23-24].

The current therapeutic options for the treatment of AD are acetylcholinesterase inhibitors, which increase neurotransmission at cholinergic synapses in the brain and reduce temporarily the cognitive deficit [25].

Cholinergic dysfunction can be associated with a decrease in the production of the neurotransmitter acetylcholine. Many approaches leading to enhancing concentration of acetylcholine at the synaptic cleft have been suggested. An understanding of the protein structure and mechanism of catalytic action of AChE is important for the design of a new potent inhibitor of AChE.

### 2.1. Tacrine

**Figure 1:**
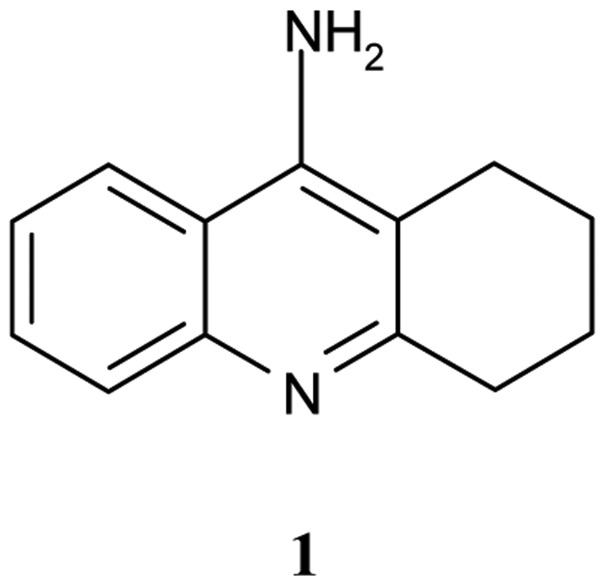
Tacrine **1**.

Tacrine, 9-amino-1,2,3,4–tetrahydroacridine (Cognex, THA, **1**, Figure 1) is a nonclassical acetylcholine inhibitor (AChEI), drug, that binds to both AChE and BuChE [5].

In 1993 tacrine was licensed in the US, Canada and parts of Europe as the first agent specifically approved for the treatment of cognitive symptoms of AD (Pfizer and Warner-Lambert, New York, NY). Tacrine is extensively metabolized by cytochrome P450 (CTP450) to at least three metabolites. The major metabolite, 1-hydroxytacrine is active [26]. Approximately 20% of tacrine-treated patients may show improvement, but its use has been limited due to the severe side effects which have been reported, e.g., hepatotoxicity and gastrointestinal antagonism, which represent important disadvantages [27,28]. It is necessary to point out that all inhibitors of AChE show gastrointestinal side effects to some degree (a consequence of their peripheral cholinergic effects). In general, AChE inhibitors can improve cholinergic transmission but cannot stop the process of neurodegeneration, however some of them are able to inhibit the apoptotic process and these AChE inhibitors possess neuroprotective effects. Use of tacrine has greatly decreased following the recent development of safer AChEIs (galantamine, donepezil, rivastigmine, and Huperzine).

Since tacrine was synthesized in 1945 [29], many analogues have been made and tested for a variety pharmacological effects. Tacrine itself was produced during an investigation into the antibacterial properties of acridine derivatives, and, although it lacks antibacterial effects, it has several other actions. Tacrine was found to antagonize the respiratory depression caused by morphine [2] before it was known to inhibit both acetyl- and butyrylcholineesterase [5] enzymes. Additional effects of tacrine were discovered later: blocking of monoamine oxidase activity [30], inhibition of neuronal uptake of 5-hydroxytryptamine receptors (5-HT), and dopamine [31], blockade of certain potassium ion channels [32], interaction with muscarinic acetylcholine receptors [33]. Various tacrine derivatives such as hydroxyl- [34] and halogenated [35] derivatives, *bis*-[36] and hetero/homodimers [37], tetracyclic tacrines [38], peptidic-tethered [39] and dihydropyridine derivatives [40] amongst other have been synthesized in an effort to mitigate the side effects concomitant with increasing efficiency. Research was focused on compounds which would simultaneously bind to active and peripheral binding sites. It was suggested that these compounds could raise levels of acetylcholine by binding to the active site, and prevent Aβ aggregation.

### 2.2. Non-Tacrine Inhibitors

**Figure 2:**
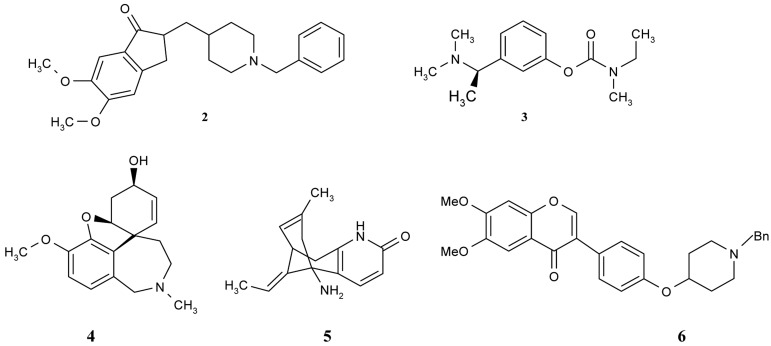
Selected AChE inhibitors-donepezil (**2**); rivastigmin (**3**); galantamine (**4**); Huperzine A (**5**); and flavonoid derivative (**6**).

Donepezil (Aricept) is another nonclassic, centrally acting, reversible, noncompetitive AChEI that was approved in 1997 for the treatment of mild-to-moderate AD and dementia (Figure 2). Its selectivity for AChE is in the periphery. When compared to tacrine, donepezil exhibits greater central nervous system (CNS) AChE selectivity, longer elimination half-life and little or no potential for hepatotoxicity. Donepezil is metabolized by cytochrome P450 2D6 (CYP2D6) and cytochrome P450 3A4 (CYP3A4) via demethylation, debenzylation, hydroxylation, oxidation to the *cis*-*N*-oxide and glucuronidation. The 6-*O*-desmethyl metabolite accounts for 11% of the dose, and exhibits AChE inhibitory activity comparable to that of the parent compounds [26,41].

Rivastigmin (Exelon) is a centrally selective, arylcarbamate AChEI that was approved in 2000 for oral administration (Figure 2). Because of the slow dissociation of the carbamylated enzyme, it has been referred to as a pseudo-irreversible AChEI. Like donepezil, rivastigmine exhibits a low level of hepatotoxicity. It is rapidly and extensively hydrolyzed in the CNS by cholinesterase with a minimal involvement of CYP450. The phenolic metabolite is excreted primarily via the kidneys [26,41].

Galantamine (Razadyne, Figure 2), which was introduced in 2001, is an alkaloid found in plants of the family *Amaryllidaceae*, which includes the daffodil (*Narcissus pseudonarcissus*) and snowflake (*Leucojum aestivum*). It is a reversible inhibitor of AChE, but it does not appear to inhibit butyrylcholinesterase. In view of the fact that it is a tertiary amine and can cross the blood-brain barrier, it is indicated for the treatment of mild-to-moderate AD and dementia. It has also been used as an anticutate agent in anesthesia. Galantamine differs from other cholinesterase inhibitors, because it allosterically binds to nicotinic receptors, giving it a dual cholinergic action. It is metabolized (75%) by CYP2D6 and CYP3A4 to create the normethyl, *O*-desmethyl and *O*-desmethylnormethyl metabolites, along with some other minor metabolites. Unlike tacrine, galantamine is not associated with hepatotoxicity [26,41].

Huperzine A, a novel alkaloid isolated from the Chinese club moss plant *Huperzia serrata*, a member of the *Lycopodium species*, is a potent, highly specific and reversible inhibitor of AChE (Figure 2). The potency and relative safety of Huperzine A rendered it a promising drug for the ameliorative treatment of AD [42]. Huperzine A temporarily blocks acetylcholinesterase, which is concentrated in the brain, spinal cord, and red blood cells. By reducing the activity of acetylcholinesterase, Huperzine A may help to reduce the breakdown of acetylcholine, thereby keeping more of it in the blood. Several studies of memory-impaired animals and humans have shown that taking Huperzine A may help to relieve dementia. This compound has already been approved in China for the treatment of AD [43-44].

Compared with tacrine, donepezil, and rivastigmin, Huperzine A has better penetration through the blood–brain barrier, higher oral bioavailability, and longer duration of AChE inhibitory action and fewer peripheral cholinergic side effects [45]. Huperzine A possesses the ability to protect cells against hydrogen peroxide, amyloid beta (Aβ) peptide, glutamate, ischemia, staurosporine-induced cytotoxicity and apoptosis.

Furthermore, there are a number of interesting compounds which belong to different groups and which also have natural origins—flavonoids. Shen *et al*. [46] synthesized, designed and evaluated a series of flavonoid derivatives as acetylcholinesterase inhibitors that could bind simultaneously to the peripheral and catalytic sites of the enzyme. Among them, some derivatives were found to inhibit the enzyme in micromolar range and isoflavone derivatives possessed more potent inhibitory activity than other flavonoid derivatives. The best compound (compound **6**, Figure 2) had its inhibitory activity in the same range as the reference donepezil. Preliminary structure-activity relationships and a molecular modeling study for this compound have revealed that the isoflavone moiety plays a key role in the interaction of this series of derivatives with AChE by acting as an anchor in its peripheral anionic site.

### 2.3. Hybrid Molecules of Tacrine-Improvement of Cholinergic Neurotransmission

Four inhibitors of AChE are known to have a positive effect on the treatment of AD symptoms—donepezil, rivastigmine, galantamine, and tacrine. These compounds were approved by the U.S. Food and Drug Administration for the treatment of AD. It has been proposed that the PAS of AChE could be somehow related to the aggregation and deposition of Aβ peptide, which is an early event in the neurodegenerative cascade of AD. These findings have led to an interest in a bi- and multifunctional drugs strategy to simultaneously inhibit ACh hydrolysis and AChE-induced Aβ aggregation. The aim of molecular designers is to develop more effective inhibitors of AChE than the current inhibitors with neuroprotective effects and with marginal side effects.

New types of inhibitors such as tacrine-based substances with dual binding site PAS and CAS have been prepared and a number of hybrid molecules of tacrine with different bioactive molecules have been designed [37,47-49]. Substances with an ability to modify activity AChE or the other proteins important for preservation of cell viability, e.g., substances with antioxidant capability or ligands of acetylcholine receptors, calcium channels, NMDA and others, have been used as bioactive molecules.

#### 2.3.1. Monotacrine inhibitors

**Figure 3:**
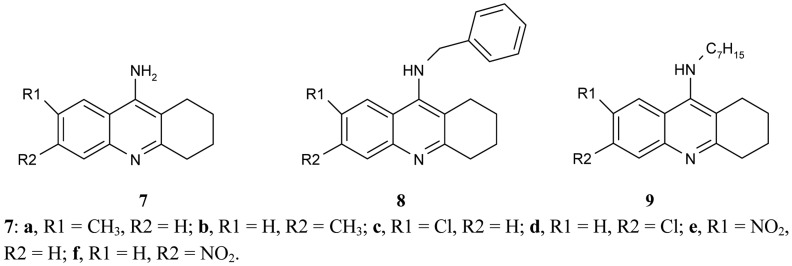
9-Amino-1,2,3,4-tetrahydroacridine derivatives **7**, **8**, **9**.

Recanatini *et al*. [50] compared the inhibitory potency of a large series of 9-aminotetrahydroacridine derivatives **7**, **8**, **9**, (Figure 3) to that of *h*AChE according to various substituents, R1, R2.

It was found that the chlorine atom, and the CH_3_ and NO_2_ groups in the 6-position (compounds **7b**, **d**, **f**) were more active AChEIs than the substituents in position 7 (compounds **7a**, **c**, **e**). The double substitution of chlorine atoms in 6- and 7- position cancels the effectivity of tacrine derivative. The results show that the biological activity of ligands depends on the electron character of the substituent (electronaceptor substituents increase and electrondonating substituents decrease inhibitory potency). The inhibitory activity of the synthesized compounds are in the range of IC_50_ = 0.0099 ± 0.0003 μM for compounds **7d**, IC_50_ = 0.028 ± 0.001 for compounds **7f**, and IC_50_ = 5.2 ± 0.1 μM. While the benzyl group decreases the AChE inhibitory potency for the 6-substituted tacrine analogues (compounds **8**) and the *n*-heptyl group, it strongly increases the activity of the 7-methyl analogue (compound **9**).

**Figure 4:**
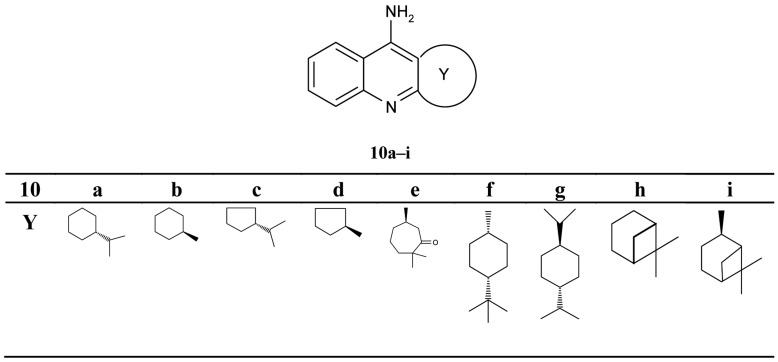
Terpenic chiral 9-aminotetrahydroacridine analogues **10a–i**.

Pisoni and his colleagues [51] focused their attention on a new series of the chiral terpenic tacrine analogues (Figure 4).

The authors discussed the configuration, the size of the ring and the bulk of substituent, and the increase and decrease of inhibiting potency in comparison with tacrine. The seven-membered analogues **10e** were found to be a significantly more potent inhibitor when compared with other compounds (IC_50_ = 0.288 μM).

**Figure 5:**
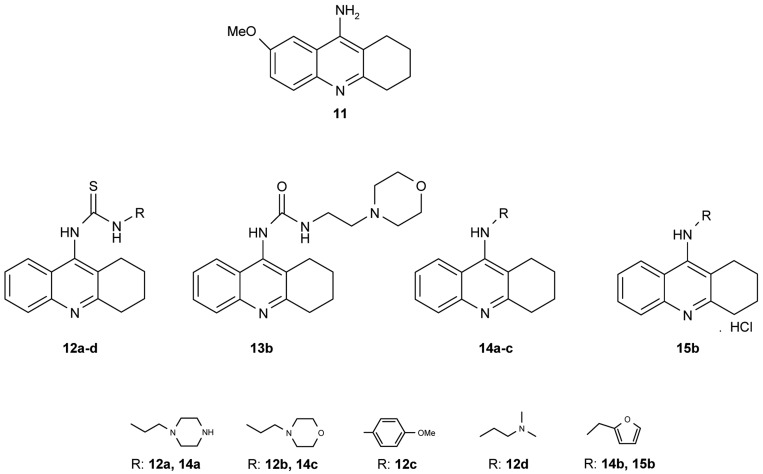
7-MEOTA **11** and novel 9-substituted tacrine congeners **12–15**.

Novel tacrine congeners (Figure 5) with side ligands suitable for optimal interaction with the peripheral and catalytic sites of acetyl- and butyrylcholinesterase have been synthesized [52] using either 9-isothiocyanato-1,2,3,4-tetrahydroacridine or 9-chloro-1,2,3,4-tetrahydroacridine.

The synthesized compounds were all tested for their ability to inhibit the activity of both AChE and BuChE. For comparison, tacrine and 9-amino-7-methoxy-1,2,3,4-tetrahydroacridine hydrochloride (7-MEOTA) [52] were used as standards. The most promising inhibition, *i.e.*, the lowest IC_50_ value, of AChE by the compounds synthesized in this work was exhibited by **14c** (IC_50_ = 0.972 μM), closely followed by **14b** and **15b** (**14b**: IC_50_ = 1.21 μM; **15b**: IC_50_ = 1.26 μM). Although compounds **14b**, **c** and **15b** failed to match the inhibition displayed by tacrine, which was cca an order of magnitude more effective, they were, in fact, more effective in inhibiting AChE than 7-MEOTA. For the inhibition of BuChE, several compounds were comparable to tacrine, **12c**: IC_50_ = 0.503 μM; **13b**: IC_50_ = 0.812 μM; **14b**: IC_50_ = 0.0204 μM; **14c**: IC_50_ = 0.164 μM and **15b**: IC_50_ = 0.0823 μM. Notably, all of the tested compounds exhibited much higher inhibitory activity towards BuChE, the only exception being 7-MEOTA. Indeed, all compounds were more effective inhibitors of BChE in comparison to 7-MEOTA. The most potent inhibitor of AChE was the morpholine aminoderivative **14c**, but, in contrast, the morpholine thiourea **12b** was the least active inhibitor. Moreover, the thiourea derivatives studied here were significantly less active. For example, the replacement of sulphur by oxygen (**12b**,**13b**) produced a compound with a three-fold increase in AChE activity and a magnitude increase in BChE activity by nearly two orders.

**Figure 6:**
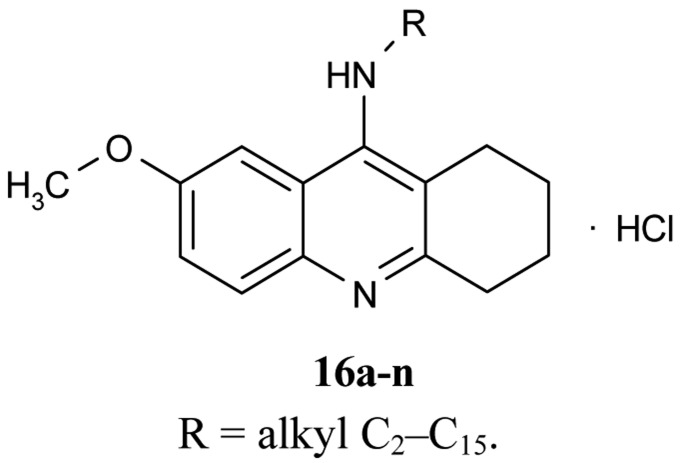
*N*-Alkyl-7-MEOTA hydrochlorides **16a–n**.

A number of papers concerning 7-MEOTA **11** as a new Czech cholinergic drug first synthesized by Patocka *et al*. [53] have also been published. In connection with the previous obtained results [54-55], fourteen new *N*-alkyl 7-MEOTA analogs hydrochlorides **16a–n** which were found to be less toxic than tacrine were synthesized [55] (Figure 6). Their activity *in vitro* on AChE and BuChE showed inhibitory ability in μM scale.

The inhibitory ability and selectivity index for *h*AChE of new compounds were compared to standards of THA, 7-MEOTA. Compound **16e** showed the best selectivity ratio for AChE (IC_50_ = 0.10 μM, which is five more potent than THA). The docking with compound **5** showed that the 7-MEOTA moiety was bound and the active gorge between Trp86 and Tyr337 by π-π stacking in the PAS anionic aromatic site.

#### 2.3.2. Dual binding tacrine inhibitors

##### 2.3.2.1. *Bis*-tacrine ligands

About ten years ago, *bis*(7)-tacrine analogues linked by an alkylene chain (*bis*(*n*)-cognitin) were prepared and it was proved that these dimeric molecules of tacrine offered a much stronger potency and selectivity towards AChE [56]. *Bis*(7)-tacrine possessed a multi-target effect including inhibition of AChE, NMDA receptors and nitric oxide synthase signaling [57]. *Bis*(7)-tacrine simultaneously binds at both the ACS and the PAS. This dimeric analogue provides a higher selectivity towards AChE over BuChE. Unfortunately, THA is not without serious toxicity, so it would be logical to develop more potent and selective inhibitors for AChE inhibition which would be similar to THA.

The synthesis of several homodimeric tacrine-based AChE inhibitors was reported [56,57], the increased inhibitory potency and target specificity of which was the result of the simultaneous binding of the units to the active and peripheral anionic sites of AChE. Consequently, tacrine homobivalent ligands could be promising drug candidates for the treatment of AD. In the search for highly selective and potent derivatives of tacrine (**1**), a number of homodimeric tacrine congeners were synthesized and tested for their effects on acetylcholinesterase (AChE) and butyrylcholinesterase (BuChE) inhibitions.

**Figure 7:**
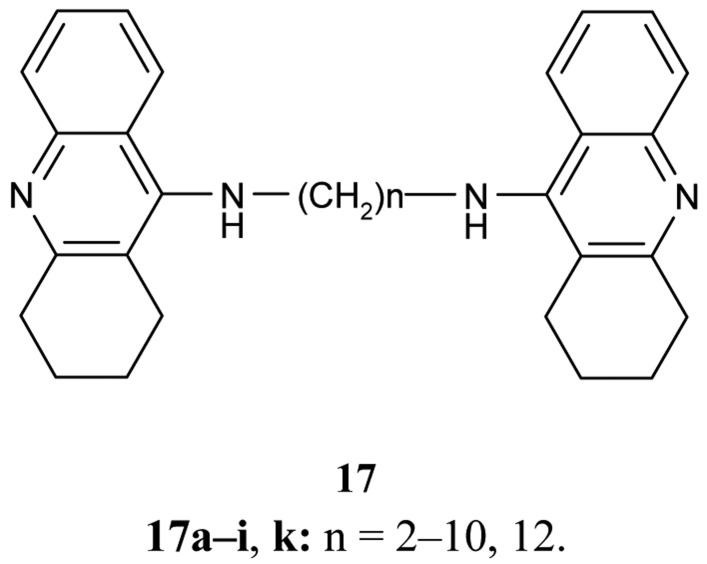
Homotacrine ligands analogs **17**.

A series of bifunctional AChE inhibitor analogs of tacrine, linked with alkylene tethers **17**, were synthesized (Figure 7). Their ability to interact with the catalytic and peripheral sites of AChE has been confirmed [58-60].

Concerning the inhibition effect of the synthesized compounds toward AChE and BuChE, it was found that the heptylene-linked dimmer **17f** shows the optimum potential activity related to the length of the alkylene chain (IC_50_ = 0.40 ± 0.025 nM, IC_50_ BuChE/IC_50_ AChE = 980) in comparison with THA [56].

Also of interest is the investigation of Carlier *et al*. [60] using *bis*-hydrochloride salts of the previously mentioned bases **17** in the series of ammonia, dimethylamine, 4-aminopyridine, 4-aminoquinoline, and tacrine. The compounds follow trends in IC_50_ values regarding their calculated desolvatation free energies ΔΔG*_theor_* in water/vacuum, and suggest the importance of ligand hydrophobicity for cation-π interaction with peripheral sites.

**Figure 8:**
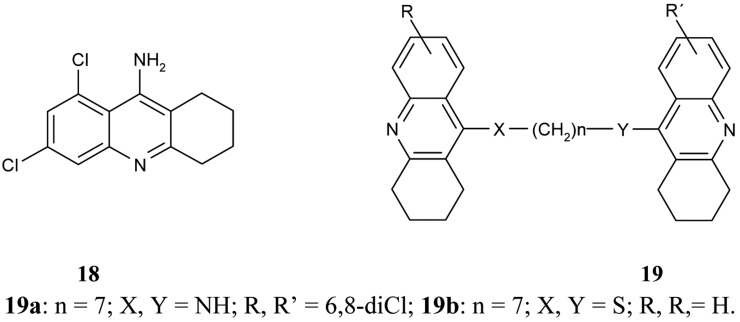
9-Amino-6,8-dichloro-1,2,3,4-tetrahydroacridine **18**, homotacrine hybrids **19**.

Following on from the above mentioned problems, Savini *et al*. synthesized a new series of homodimeric tacrine analogs **19** (Figure 8), which are characterised by a different length and character of the tether [61]. The most potent AChEI was homodimer 6,8-dichlorotacrine **19a**.

**Figure 9:**
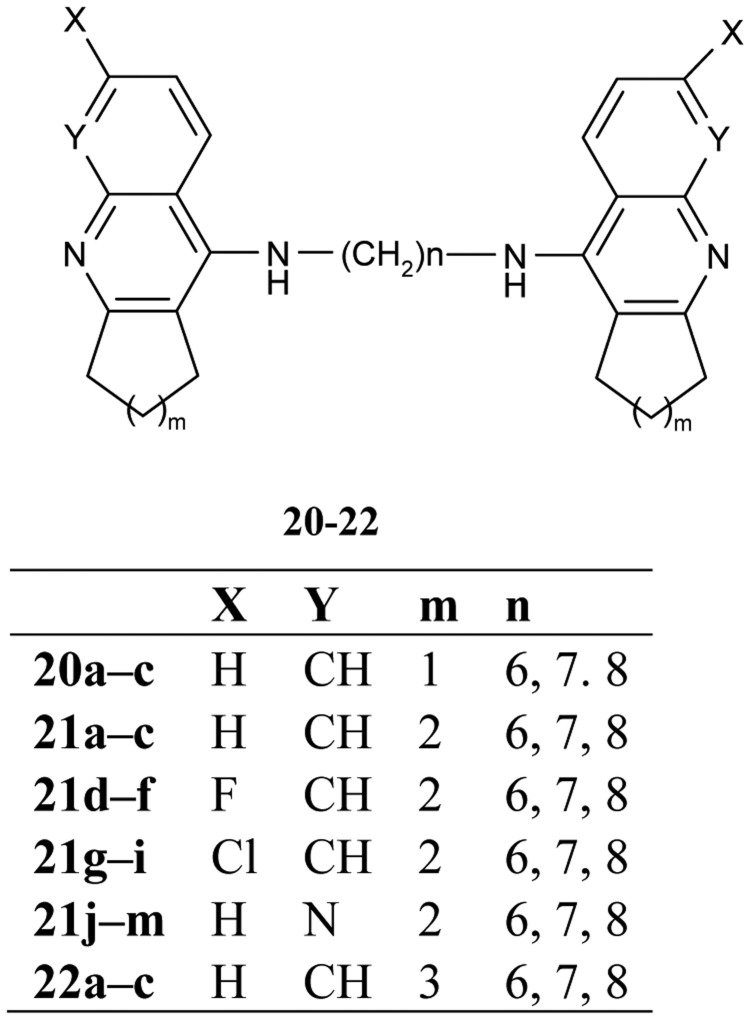
*Bis*-tacrine congeners **20–22**.

The congeners of tacrine-tacrine homodimers with 6–8 methylene units **20–22** (Figure 9) were observed by Hu and his colleagues [62]. Their studies integrated previous findings to carry out changes in carbocyclic ring size on the 6-substituted position and isosteric modification toward enhanced optimization for AChE inhibition potency.

The work describes in detail the influence of various structural changes, such as the size of the carbocyclic ring **21a–m**, **22a–c**, incorporation of a halogen at the 6-position of homodimeric tacrines **21d–i**, insertion of an aza into the tacrine nucleus **21j–m** and the length of tether inhibition potency of AChE and BuChE. The homodimeric congeners, heptylene-linked *bis*-(6-chloro)-tacrine **21h** showed much improved potency and selectivity of AChE (IC_50_ = 0.07 nM, BuChE/AChE = 371).

##### 2.3.2.2. Heterotacrine ligands

A great attention was dedicated to new AChEI compounds, with dual binding mode of action, which bear a tacrine-related unit for interaction with the active site, either at the mid-gorge and peripheral site. These hybrid molecules were potent and selective inhibitors of human AChE, and they were able to interfere with both formation and amyloid aggregation of the Aβ peptide. A number of AChE inhibitors have been identified in plant extracts, and one of them is Huperzine A. Hybrid molecules of tacrine with Huperzine A had been designed and studied during the last decade. Several hybrid molecules of tacrine with Huperzine A were prepared by Badia *et al*. [63] and Camps *et al*. [64,65], and some of them were more active than tacrine.

**Figure 10:**
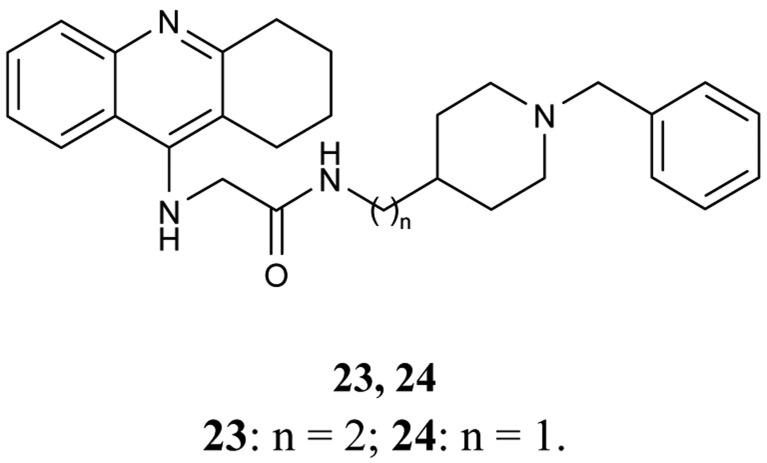
Tacrine-donepezil hybrids **23**, **24**.

Donepezil as a well known drug in the treatment of AD, and is still the focus of research to find new potent AChE inhibitors. In this context, Shao *et al.* [66] prepared novel tacrine-donepezil hybrids as dual binding side AChE inhibitors. Both hybrids **23** and **24** (Figure 10) were found to be more potent for AChE inhibition than tacrine.

*In vitro* AChE and BuChE inhibitory activities of the hybrids **23** and **24** were IC_50_ = 6.0 nM, IC_50_ = 10.2 nM, respectively.

**Figure 11:**
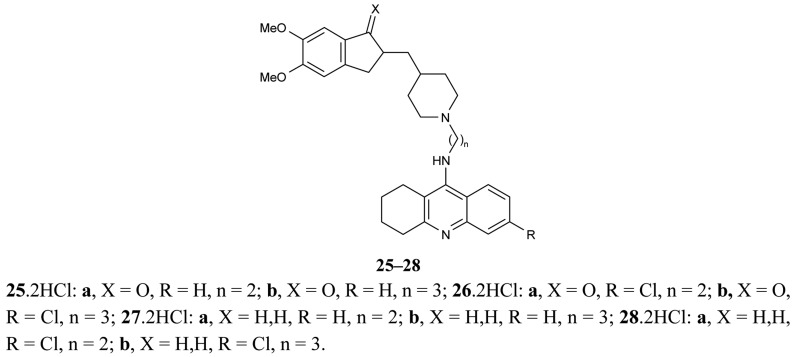
Tacrine-donepezil hybrids **25–28**.

Another paper concerning the above mentioned topic of donepezil-tacrine hybrids **25-28** was published by Camp *et al*. [67], in which the hybrids were seen to interact simultaneously with the active, peripheral and midgorge binding sites of AChE (Figure 11). These compounds contain 5,6-dimethoxy-2-[(4-piperidinyl)methyl]-1-indanone moiety of donepezil with tacrine or 6-chlorotacrine as congeners, and have been tested for their ability to inhibit AChE and BuChE.

The highly potent compound **26b** bearing an indanone unit chlorine atom at the tacrine unit and a tether length of three methylenes were tested against bovine and human AChEs and exhibited IC_50_ values in the subnanomolar range.

**Figure 12:**
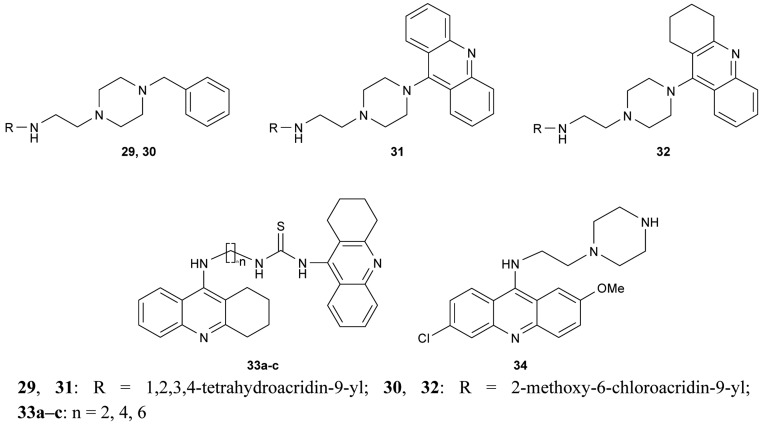
Heterodimeric tacrine/acridine hybrids **29–34**.

Novel bivalent tacrine/acridine cholinesterase inhibitors (**29–32**, **33a–c**, **34**, Figure 12) showing dual binding affinity toward AChE and BuChE were synthesized using 9-chlorotetrahydroacridine, 9-chloroacridine, 6,9-dichloro-2-methoxyacridine and 9-isothiocyanatotacrine as synthons. Their neuroprotective activity against *h*AChE and *h*BuChE reached nanomolar concentrations [68-70].

**Figure 13:**
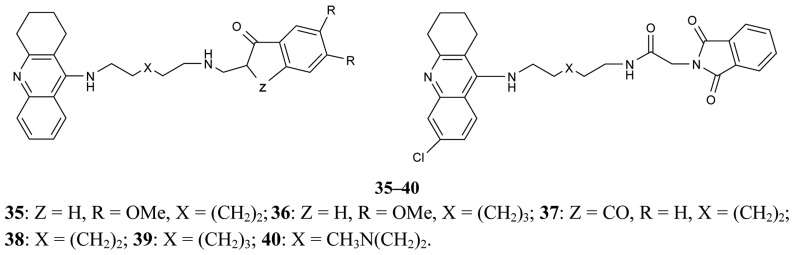

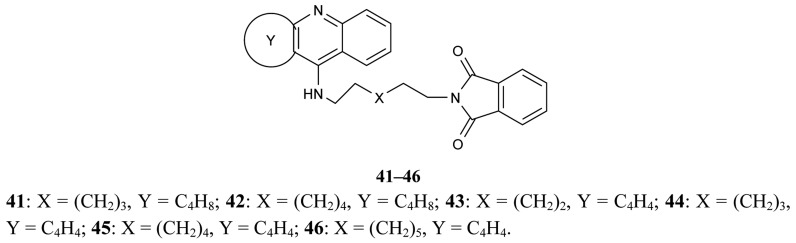
Tacrine-donepezil hybrids **35–40** and **41–46**.

A new series of donepezil–tacrine hybrid derivatives **35–40** and **41–46** (Figure 13) combining a tacrine, 6-chlorotacrine or acridine unit were synthesized by Alonso *et al*. [71]. These derivatives obtained a tacrine heterocyclic ring as AChEI and indanone or relative heterocycles responsible for the binding to the peripheral site of the enzyme.

**Figure 14:**
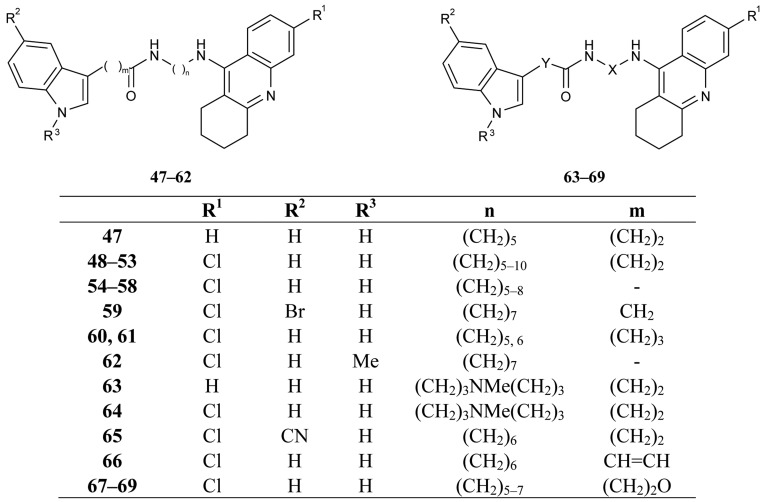
Tacrine-indole heteroligands **47–69**.

These derivatives are able to bind simultaneously to both sites of the enzyme. A propidium competition assay confirms the interaction of the compounds **35–46** at the PAS of AChE. Compounds **40** (IC_50_ = 2.8 nM), and **46** (IC_50_ = 2.4 nM) were found to be the most potent AChEI, while tether length for the linker seemed to be 9 (compound **46**) or 10 (compound **40**). The most active AChEIs discovered to date are represented by a large series of novel tacrine-indole heteroligands **47–69** synthesized by Muñoz-Ruiz [72] and his colleagues (Figure 14). The dual binding mode of the compounds under study takes place at both the catalytic and peripheral sites of the enzyme.

The highest AChE inhibition activity was shown by hetrodimers **49** and **50**, bearing two methylene groups between the indole ring and the amide functionality and the tether length from the amide to the tacrine unit bearing 6 or 7 methylenes (**49**: IC_50_ = 70 pM; **50**: IC_50_ = 20 pM).

**Figure 15:**
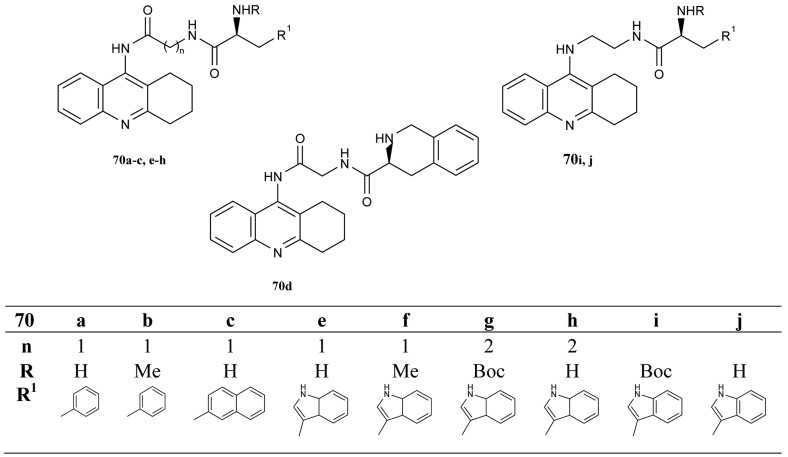
Tacrine heteroligands containing peptidic tethers **70a–j**.

The next interesting series of heterotacrine ligands as AChEIs containing peptidic tethers and one or two tacrine skeletons (**70a–j**) were synthesized [39] by Butini (Figure 15). The individual sections of the studied compounds were coupled with natural or unnatural l-amino acids, hydrophobic in nature, chosen on the basis of adaptability to the cleft (l-glutamate and l-proline). The compounds containing tryptophane units **70i** and **70j** are more potent and selective *h*BuChEIs (IC_50_ = 1.87 nM; IC_50_ = 1.33 nM, respectively).

**Figure 16:**
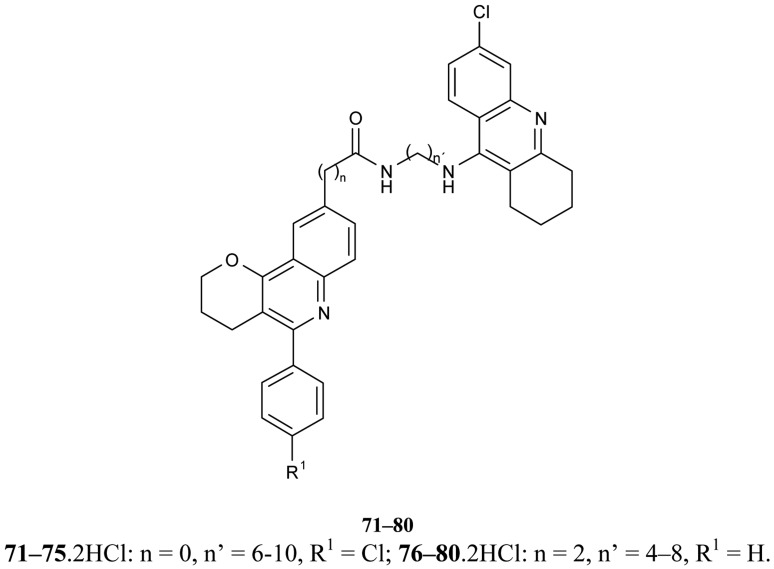
Heterotacrine-quinoline hybrids **71–80**.

New hybrids of compounds consisting of tacrine or 6-chlorotacrine units as the active sites of interacting moiety, either the 5,6-dimethoxy-2-[(4-piperidinyl)methyl]-1-indanone fragment of donepezil, or a 5-phenylpyrano[3,2-*c*]quinoline system, as the peripheral site interacting unit (Figure 16) **71–80** were reported [64]. All of the studied compounds demonstrate more potent AChEI than tacrine; 6-chlorotacrine and donepezil the best of the synthesized compounds are **81–88** (IC_50_ = 14.4–19.2 nM). A possible binding mode of studied ligands can be explained by molecular docking studies.

**Figure 17:**
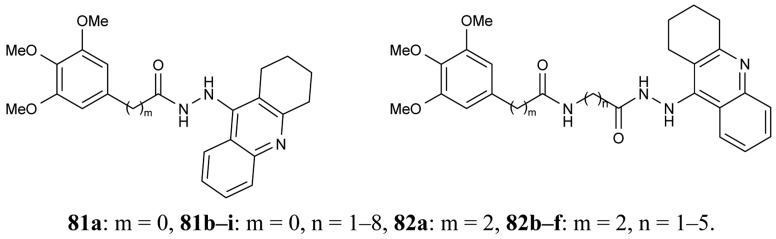
Trimethoxybenzene-tacrine hybrids **81** and **82**.

In order to obtain novel inhibitors of AChE and BuChE, a series of heterodimer derivatives **81**, **82**, in which the tacrine moiety was connected to the trimetoxybenzene unit through a hydrazide-based linker of varying length were synthesized by Elsinghorst [73] (Figure 17).

From the data retrieved for acetylcholinesterase, a comparison of the corresponding derivatives of both series revealed that in most cases compounds **81** were superior to compounds **82**. It was proved that the most potent heterodimeric inhibitors contain an 11- or 12-atoms spacer (**81h**, IC_50_ = 3.25 nM; **81i**, IC_50_ = 2.73 nM). In the case of the other series, **82f**, with a total spacer length of 11 atoms ligand, seems to be the best inhibitor.

##### 2.3.2.3. Multifunctional tacrine ligands

**Figure 18:**
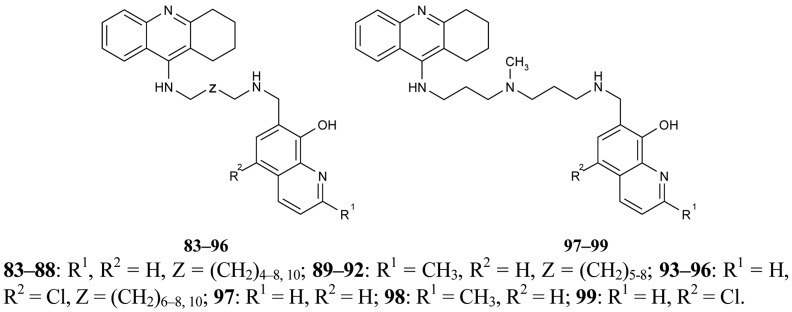
Heterodimeric tacrine-quinoline derivatives **83–99**.

In recent years, several works concerning active research into multifunctional agents with different complementary biological activities to AD have been published. Novel multifunctional compounds **83–99** with antioxidant, metal-binding properties, inhibition of Aβ aggregation and dual inhibition of AChE/BuChE have been designed and synthesized by Fernández-Bachiller *et al*. [74] (Figure 18). These tacrine-8-hydroxyquinoline (PBT2) hybrids show excellent inhibition activity against *h*AChE and *hBuChE* at nanomolar and subnanomolar concentrations. The most active derivative was compound **86** which contains unsubstituted 8-hydroxyquinoline fragment and a methylene tether of 7–10 carbons (IC_50_ = 20 nM). Three of the synthesized compounds **86**, **90**, **94** were chosen for evaluation due to their characteristics as free radical scavengers, their antioxidant activities and their inhibition of Aβ aggregation.

Further interesting work has been dedicated to multifunctional compounds of *bis*-tacrine derivatives which simultaneously interact with catalytic and PAS of the enzyme, and which are able to inhibit AChE induced Aβ aggregation and acting as metal chelators (Bolognesi *et al.* [75]).

**Figure 20:**
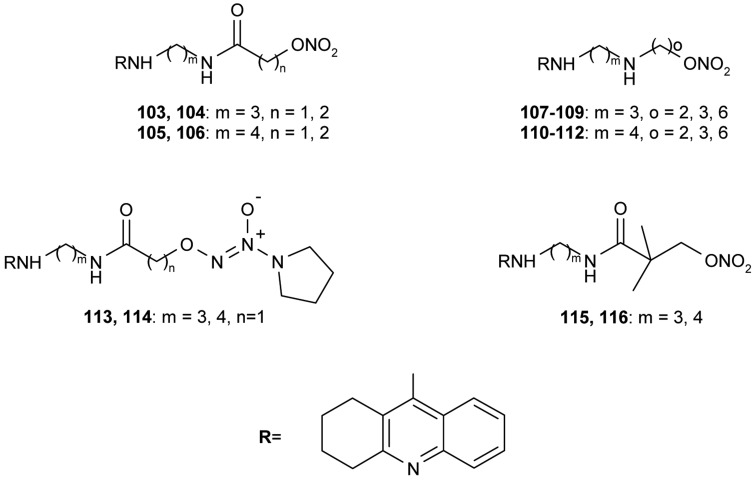
NO-donor-tacrine hybrids **103–116**.

All synthesized hybrids **100–102** show dramatically more potent inhibition of AChE than tacrine. To improve the hepatotoxicity of tacrine hybrids, novel amine and amide-linked nitrate- and NOate-tacrine hybrids **103–116** (Figure 20) have been synthesized in connection with their ability to inhibit cholinesterases and for their vasorelaxation effects [76]. The most active target compounds were **108**, **111** with high AChEI (IC_50_= 6.4 nM, 5.6 nM) and BuChE (IC_50_= 5.5 nM, 9.9 nM).

**Figure 21:**
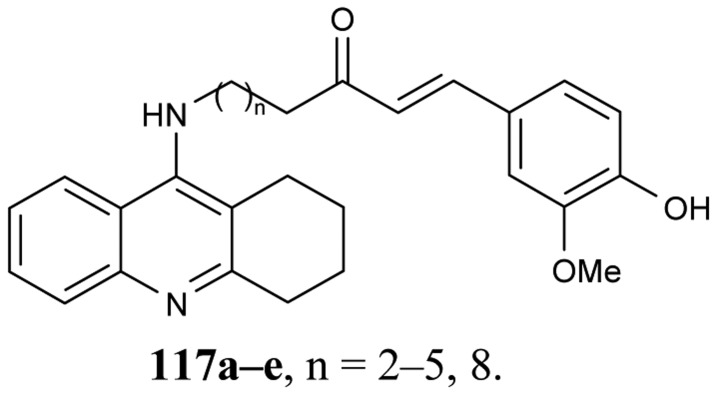
Tacrine-ferulic acid hybrids **117a–e**.

A new series of tacrine-ferulic acid hybrids **117a–e** with antioxidant effects have been synthesized and tested as multipotent anti-Alzheimer drug analogs by Fang *et al*. [77] (Figure 21). Among all of the tested hybrids **117a–e**, ligand **117d** showed a greater activity toward AChE/BuChE (IC_50_ = 4.4 nM, 6.7 nM, resp.) in comparison to tacrine. The optimal differences between the tacrine-like heterocycle and the ferulic acid moiety were 6–7 atoms long.

**Figure 22:**
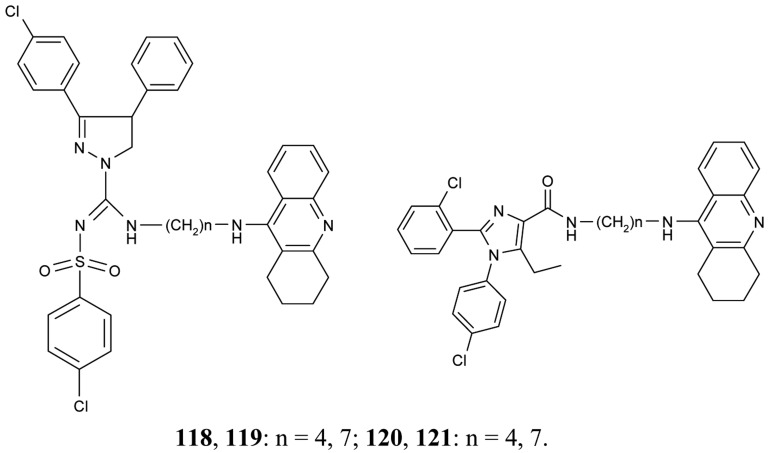
Tacrine-CB_1_ receptor antagonists **118–121**.

To improve aqueous solubilities of tacrine ligands in pharmacological testing, a novel series of AChEIs **118–121** bearing tacrine pharmacophore and two different CB_1_ antagonistic pharmacophores with a polar group or ionizable nitrogen atom have been synthesized; **118** and **119** contain pyrazoline rings, while **120** and **121** contain imidazole rings (Figure 22) [78].

The imidazole derivative **120** with spacer length n = 4 showed high CB_1_ receptor affinity (48 nM) and demonstrated AChE inhibitory activity at the same potency as tacrine.

**Figure 23:**
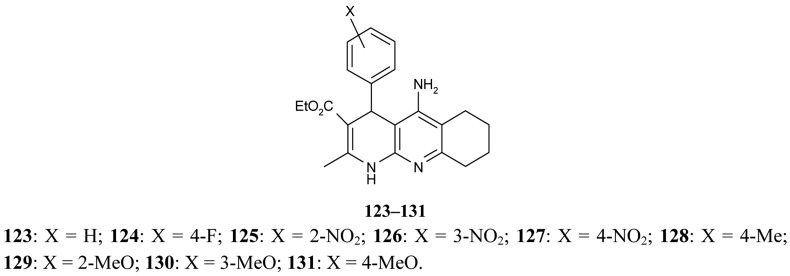
Tacrine-1,4-dihydropyridine hybrids **123–131**.

Marco-Contelles [79] have published their results on the synthesis of novel multipotent tacrine-1,4-dihydropyridine hybrids **123–131**. The multipotent character of the compounds under study is concerned with their AChE/BuChE inhibition, propidium iodide displacement, Ca^2+^ channel blockade, and neuroprotective activity (Figure 23).

Their results confirm that compounds **123–131** are very selective and potent AChEIs and that they show outstanding neuroprotective profiles and a moderate Ca^2+^ channel blockade effect.

## 3. Tacrine and Anti-Amyloid Aggregation

Amyloid aggregation of proteins is a complex process associated with amyloid-related neurodegenerative disorders such as Alzheimer's and prion diseases. The conversion of a specific protein or protein fragment from soluble native states into amyloid aggregates results in the formation of amyloid deposits with a single predominant protein compound characteristic of each disease. In AD, two types of aggregates have been identified in the brain: intracellular neurofibrillary tangles created by tau protein and extracellular senile plaques NFTs consisting of Aβ peptide. Tau protein is also involved in various tauopathies. The fibrillar form of huntingtin is associated with Huntington's disease, and transthyretin with familial amyloid polyneuropathy. Amyloid deposits formed by alpha-synuclein are found in patients with Parkinson's disease [80].

The formation of amyloid aggregates is triggered by nucleation-dependent polymerization from subunit protein by axial stacking of beta strands, generating a cross-beta-sheet structure at the core of the filaments [81]. The process of aggregation involves a series of steps during which many intermediate aggregation states are populated. Recent evidence points to these intermediate states as the toxic moieties primarily responsible for cell damage or cell death, which are critical steps in the origin and progression of amyloid-related neurodegenerative disorders [82]. Recently, growing evidence has emerged that soluble oligomers may be the main toxic species [83,84]. The reason why early aggregates are more toxic than mature amyloid fibrils is not yet clear, although it is likely that it arises from the accessibility of oligomers to interact inappropriately with cellular species leading to deregulation of cell homeostasis [85-87].

Currently, the precise mechanisms of toxicity have not been fully elucidated; however, there is evidence that reduction of amyloid deposits lead to alleviation of disease's symptoms [88,89]. As a consequence, considerable effort has been directed toward the identification of the substances able to reduce amyloid aggregation. In the past few years, a range of diverse small molecules have been found to inhibit or reduce the amyloid aggregation of various proteins, particularly in relation to Aβ deposition, the aggregation of transthyretin and the formation of protease-resistant forms of the prion protein [90-94]. Kim [93] investigated the effect of several classes of naturally occurring compounds on Aβ amyloid aggregation. The results of his study showed that curcumins, flavones type flavonoids, and naphthoquinones are potent inhibitors of Aβ fibrillization formation *in vitro.* Doplhin *et al*. [94] demonstrated that longer trioxime oligomers showed an effective reduction of Aβ amyloid fibrils formation. One of the first compounds to be identified with blocked tau-tau interaction was the phenothiazine methylene blue [95]. This compound was shown to partially disrupt the structure of isolated paired helical filaments and could affect tau fibrillization. Interestingly, this dye molecule has now progressed into clinical testing in AD patients [96]. In a screen of 42 compounds, Taniguchi *et al*. [97] identified small molecules categorized as phenothiazines, polyphenols and porphyrins which were able to inhibit tau amyloid polymerization. It was found that a number of anthraquinones were able to inhibit tau aggregate formation and could also induce disassembly of pre-formed tau fibrils. A similar effect was observed for cyanine dyes [98] and rhodamines [99].

Acridine-based compounds were identified as potent inhibitors of protease-resistant forms of prion protein [100]. Anti-scrapie activity, probably through inhibition of the formation of protease-resistant prion protein, has been found also for other acridine derivatives [92,101]. It has been shown that some acridines and *bis*-acridine derivatives are able to reduce scrapie prion concentration in infected cells [102]. The first acridine derivatives reported to inhibit or interact with the Aβ polymerization process are diaryl heterocyclic compounds and 9-acridone derivatives [103,104]. More recently, a high affinity for Aβ aggregation was detected for fluorinated acridine orange analogue [105,106]. The inhibiting activity was observed for acridine derivatives, namely for quinacrine and quinacrine mustard [107]. They have shown that these compounds are able to inhibit formation of amyloid fibrils of tau and Aβ peptide.

The tacrine dimers **100-102** characterized by AChE inhibitory activities were tested for their ability to inhibit AChE-induced Aβ fibrillogenesis [75]. In particular, 100 μM inhibitors inhibited Aβ aggregation from 53% to 76%, **100** being the most potent and slightly more potent than **17f** (76% compared to 68%). These findings suggest that effective inhibitor concentration in the aggregation assay is much higher than the IC_50_ values in comparison to the enzyme. Muñoz-Ruiz *et al*. designed dual binding site acetylcholinesterase inhibitors as potent new drugs inhibiting amyloid Aβ peptide aggregation through binding to both catalytic and peripheral sites of the enzyme [72]. The heterodimers showing the best AChE inhibitory potency (**49**, **50** and **58-60**) were selected to assess their ability to inhibit Aβ peptide (Aβ_1–40_) aggregation. Results showed that the heterodimers inhibited the Aβ peptide aggregation at 100 μM in a range varying from 49% to 63%, and that they were at least as potent as propidium, which caused 46% inhibitions. The most active compounds were **49** and **50**, followed by **58**, **59**, **60** the latter being around 2-, 3- and 10-fold less active than the former compounds, respectively.

Hybrids **25-28** and **71-81** result in a significant *in vitro* ability to inhibit the *h*AChE-induced aggregation of Aβ peptide, with these hybrids exhibiting percentages of inhibition of 23–46%, 38–66%, respectively, at 100 μM. In the first series, the separation of two methylenes between the amido group of the linker and the pyrano[3,2-*c*]quinoline system leads to a higher Aβ antiaggregating effect. In the second series, the substitution pattern leading to an optimal Aβ antiaggregating effect involves the presence of an indane moiety irrespective of the length of the linker and the substitution at the tacrine unit. These results suggest that the new compounds could bind to the AChE-PAS and could therefore inhibit the Aβ fibril formation promoted by this enzyme.

Recently, we have examined a small group of structurally distinct acridine derivatives which differ in molecule planarity and bulky side groups at the middle ring moiety for their ability to inhibit lysozyme fibril formation *in vitro* [107,108]. We have found that planar acridine compounds are very effective inhibitors, while spiroacridines have been ineffective in inhibiting fibril formation. Tetrahydroacridines have had no significant effect on the prevention of lysozyme fibrillization; moreover, in the presence of some derivatives, an enhanced degree of aggregation has been detected. Anti-amyloid activity has also been observed for glycosyl acridines [109]. The different activities of the acridine derivatives studied have indicated that the structure of the acridine side chains and planarity of the acridine cyclic core are the crucial elements in determining the extent of amyloid aggregation. The highest inhibiting activity among screened compounds at all was have been detected for dimeric acridine. A similar increase in anti-amyloid activity for *bis*-acridines was observed by May *et al*. [102]. They found that covalent acridine dimers could be more potent than monomeric equivalents in decreasing scrapie-prion concentration. Dolphin *et al*. [110] investigated the potential of a multimeric quinacrine derivative to inhibit Aβ fibril formation in comparison to monomer quinacrine. The multimeric conjugate exhibits a cluster of four quinacrine derivatives on a rigid cyclopeptide core. Their data showed that the multimeric compound substantially enhanced inhibition of Aβ polymerization into amyloid aggregates. It suggests that assembling multiple copies of acridine moieties to a central scaffold allows multiple interactions with protein leading to effective anti-amyloid activity of the compound.

## 4. Neuroprotectivity of Tacrine

The typical role of AChE is to terminate cholinergic neurotransmission, but AChE has also been observed in non-neuronal tissues (erythrocytes, megakaryocytes and the others), and there is significant evidence for additional noncatalytic functions of AChE. The level of this enzyme changes during proliferation, neurite growth, apoptosis, differentiation and also the expression of AChE is changed at neurodegenerative diseases [111-116]. Cholinergic neurons in brains affected by AD express more AChE than other neuron types and it has been confirmed that these neuronal cells have higher levels of AChE-S isoform. Apoptosis has been proposed to explain the loss of the cholinergic neurons but the exact mechanisms which regulate the expression of AChE during apoptosis have not been known. Recently, it has been confirmed that up-regulation of AChE-S is associated with activity of GSK-3 which plays a central role in ER-stress induced activation of caspase 3 [117]; as a result, the inhibition of caspases has been suggested as a therapeutic strategy in neurodegenerative diseases, although the possibility of caspase-independent death means that caspase inhibition can offer only transient protection. The search for new inhibitors of AChE has become more intense since AChE was characterized as a multifaceted protein, and also since the discovery of AChE in AD plaque formation and in the neurofibrillary tangles and the amyloid-positive vessels. Even when AChE-peptide was added at nanomolar concentrations, cells died *via* an apoptotic pathway [118-121]. In light of the non-classical role of AChE, AChEIs could act as multifunctional agents and some of them could possess neuroprotective effects in addition to their AChE-inhibiting action. Therefore the development of new AChEI, including derivatives of tacrine is aimed not only at improving selectivity for AChE and the better side effect profile/low cytotoxicity, but also the potency of their their neuroprotective [49,50,122-126]. Oxidative stress is an early event in AD pathogenesis and therefore new hybrid molecules of tacrine with antioxidant capacities are being synthesized and their neuroprotective effect studied. The neuroprotectivity of new derivatives of tacrine which inhibit Aβ aggregation and the state of intracellular concentration of Ca^2+^ in neuronal tissue are also being researched.

### 4.1. Hybrid Molecules of Tacrine and Oxidative Stress

Increased oxidative stress resulting from free radical damage to cellular function can be involved in events leading to AD. New hybrid molecules of tacrine with antioxidants have been prepared to inhibit AChE and simultaneously to protect against oxidative stress. In addition, the hepatotoxicity of tacrine was confirmed and this issue could be reduced through the use of hybrid molecules of tacrine with antioxidant effects. Such types of bi-functional molecules, tacrine-8-hydroxyquinoline hybrids have recently been synthesized by Fernandez-Bachiller *et al*. [74]. As mentioned above, 8-hydroxyquinoline derivative is known antioxidant acting through the complexation of redox-active metals. These novel hybrid molecules at nano- and subnanomolar concentrations inhibited AChE, and they could be able to inhibit Aβ aggregation promoted by AChE. It was shown that tacrine-8-hydroxyquinoline hybrids possessed better antioxidant properties than Trolox and showed low cytotoxicity. Rodrigues-Franco *et al*. [127] and Fernandez-Bachiller *et al*. [128] used melatonin for the preparation of new hybrid molecules of tacrine. These hybrids were selective inhibitors of human AChE and they exhibited antioxidant capacities. Fang *et al*. [77] synthesized tacrine-ferulic acid hybrid and demonstrated that the cytotoxicity of this hybrid molecule was significantly decreased.

In neuronal cells, NO controls the release of neurotransmitters and is involved in synaptogenesis, synaptic plasticity, memory function, and neuroendocrine secretion [129]. NO might also play a protective role in AD. *Bis*(7)-tacrine is able to reduce glutamate-induced NO generation [130] and Li *et al*. [131] showed that the neuroprotection of *bis*(7)-tacrine can be mediated through the inhibition of neuronal nitric oxide synthase (nNOS) and through the moderate blockade of NMDA-receptors, as this receptor plays a role in inhibiting endogenous NOS. Kinetic studies have confirmed that *bis*(7)-tacrine selectively inhibits the NOS in a competitive manner. NO-donor-tacrine hybrids have been synthesized during research into the development of safer anti-Alzheimer drugs [132], and these hybrid molecules inhibited cholinesterases and some showed much less hepatotoxicity than tacrine in addition to possessing vasorelaxation effects.

### 4.2. Hybrid Molecules of Tacrine and Inhibition of Aβ Amyloid Aggregation

The death of neuronal cells is associated with the development of of amyloid aggregation (Section 3). Under normal conditions, Aβ peptides are degraded by the lysosomes after autophagosome/endosome fusion. It has recently been demonstrated that Aβ peptides are toxic for mitochondria and that Aβ peptides are transported into mitochondria via the mitochondrial translocase of the outer membrane machinery. Genes related to mitochondrial energy metabolism and apoptosis are upregulated before and during the appearance of Aβ plaques. It has also been suggested that autophagic insufficiency results in an accumulation of dysfunctional mitochondria and aberrant protein aggregates [133-138]. In addition to its catalytic function, AChE can also bind to Aβ peptide and act as a promoter of amyloid Aβ formation. This action is associated with PAS of AChE and such AChEI which bind to PAS could retard this aggregation.

Apoptosis is a major form of neuronal cell death and the link between autophagy and diseases associated with protein aggregation has been confirmed, although the trigger of this cellular misbalance is not yet known. Autophagy is an adaptation process, and if this process is defective than the suitable conditions for intracellular Aβ accumulation and cell death are created [139-144]. It is known that autophagic process can proceed to apoptosis. Apoptosis and autophagy share some similar hallmarks, e.g., calpains are activated during these processes but caspases are activated only during apoptosis. The action of an AChE on the autophagy of neuronal cells has not been clarified and multifunctional AChEIs could help to explain the action of AChE on cell death. Escobar-Khondiker *et al.* [144] showed that mitochondrial disturbances lead to a decrease in ATP levels that could induce Aβ misfolding. The interaction between Aβ peptide and beclin1 is not clear. Externally added Aβ peptides decreased mitochondrial function and also induced a strong autophagic response. Furthermore, the inhibition of autophagosome formation in Aβ treated cells significantly enhanced its toxicity.

The neuroprotective effects of tacrine hybrids could be associated with the inhibition of AChE-induced Aβ aggregation and inhibition of β-secretase. Two isomeric series of dual binding site acetylcholinesterase inhibitors have recently been designed and synthesized by Camps et al [64]. These hybrids, consisting of a unit of 6-chlorotacrine and pyrano[3,2-c]quinoline, possessed the potent and selective human AChE inhibitory activity and exhibited a significant *in vitro* inhibitory activity toward the AChE-induced and self-induced Aβ aggregation and toward β-secretase, in addition to the ability to enter the central nervous system.

### 4.3. Hybrid Molecules of Tacrine and Changes of Calcium Transport

Calcium is a second messenger and intracellular Ca^2+^ plays an important role in the modulation of cellular death. Overload of Ca^2+^ has even been suggested to be the final common pathway of all types of cell death [145-147]. Critical role for calcium dysregulation in the pathogenesis of AD has been discussed [148-151]. Zhu *et al*. [150] showed that cytosolic Ca^2+^ plays a key role in AChE regulation during apoptosis. The concentration of cytosolic Ca^2+^ has an important role in the modulation of apoptosis, and AChE expression during apoptosis is related to Ca^2+^ mobilization. It has been suggested that calcium dysregulation can implicated as a major contributor to neuronal cell death in AD. Green and LaFerla [147] have confirmed that calcium homeostasis was perturbed in dendritic spines adjacent to amyloid plaques. The neuroprotectivity of AChE inhibitors could be associated mainly with their action on the intracellular accumulation of Ca^2+^. Their direct effects on voltage-dependent Ca^2+^ channels, *n*AChRs (nicotinic acetylcholine receptors) or NMDA (*N*-methyl-d-aspartate) receptors have been studied very intensively. It has been confirmed that tacrine is able to inhibit voltage-dependent Ca^2+^ channels in dorsal root ganglion cells [152] and also to interact with nicotinic receptors [153-155].

Several series' of tacrine derivatives have been synthetized and their biological activity as inhibitors of AChE as well as modulators of voltage-dependent Ca^2+^ channels and nicotinic receptors has been evaluated [153-155]. Some of the tacrine hybrid molecules exhibited neuroprotectant effects and inhibited AChE. Recently Marco-Contelles *et al.* [156] prepared tetracyclic tacrine, tacrine-dihydropyridine hybrids to achieve a blockade of Ca^2+^ entry through neuronal voltage-dependent Ca^2+^ channels. They showed that tacripyrines were selective and potent AChE inhibitors in the nanomolar range and that these compounds were able to inhibit the proaggregating action of AChE on the Aβ. Tacripyrines showed a moderate Ca^2+^ channel blocking effect.

A significant loss of *n*AChR and certain types of muscarinic ACh receptors in brains affected by AD has been confirmed [157,158]. Tadaka-Takori *et al.* [159] studied the mechanism of neuroprotectivity of acetylcholinesterase inhibitors, including tacrine which protect neurons from glutamate-induced neurotoxicity in the primary culture of rat cortical neurons. They concluded that AChEI are able to induce up-regulation of nicotinic receptor expression levels.

The NMDA receptor is a key player implicated in the regulation of learning and memory and it was supposed that that one of the targets of AChE inhibitors involved in cognitive process is the NMDAR-mediated synaptic response in prefrontal cortex neurons [160]. *Bis*(7)-tacrine is a noncompetitive antagonist of NMDA receptors that can prevent glutamate-induced damage to hippocampal neurons [161]. Fang *et al.* [162] confirmed that *bis*(7)-tacrine prevented glutamate-induced cell death. They confirmed the anti-apoptosis effects of *bis*(7)-tacrine and their results showed that *bis*(7)-tacrine had neuroprotective effects against glutamate-induced RGCs (retinal ganglion cells) damage *in vitro* and *in vivo*. Luo *et al.* [163] studied analogues of *bis*(7)-tacrine and demonstrated that *bis*(propyl)-tacrine inhibited AChE and acted as a γ-aminobutyric acid subtype A receptor antagonist, and an antagonist of NMDA receptors. In cultured rat hippocampal neurons, *bis*(propyl)-tacrine voltage-dependently, selectively, and moderately inhibited NMDA-activated currents. The inhibitory effects of this AChEI increased with the rise in NMDA and glycine concentrations. Under glutamate-mediated pathological conditions, *bis*(propyl)-tacrine, in contrast to *bis*(7)-tacrine, prevented excitotoxicity with increasing effectiveness against escalating levels of glutamate and protected against middle cerebral artery occlusion-induced brain damage much more effectively than memantine.

AD is characterized by the depletion of *n*AChR. Many studies have demonstrated that the persistent stimulation of certain subtypes of *n*AChRs, such as α4 and α7, protect against neurotoxicity induced by glutamate and Aβ. The AChEIs, including tacrine, are able to protect against neuronal death induced by moderate glutamate treatment, and although it is known that *n*AChR subtypes are involved in tacrine-induced neuroprotection, the intracellular mechanisms involved are not clear [126,158]. Tacrine or its analogues are able to inhibit the apoptotic process, although they act indirectly, through their effect on Ca^2+^ homeostase. Recently, the hypothesis that AChE play a role in apoptosis has been made on the basis of information that this enzyme was first detected in the cytoplasm in the early stages of apoptosis and at later stages in the nucleus [164,165]. Park *et al.* [166,167] proposed that AChE even can assemble apoptosomes with apoptotic protease-activating factor-1 (Apaf-1) and cytochrome c, and activate caspase 9. If apoptosis was induced, the silencing of the AChE gene blocked the interaction between cytochrome c and Apaf-1. It was shown that AChE interacted with caveolin-1 in cells undergoing apoptosis. This association of AChE with caveolin-1 is required for the interaction between AChE and cytochrome c. The interaction between AChE and caveolin-1 could be indispensable for apoptosome formation. The interaction of AChE with other apoptotic molecules during apoptosis is not completely clear. Recently, Ye *et al.* [168] studied the relationship between AChE and classical apoptotic molecules during apoptosis, and confirmed that AChE-S plays a pro-apoptotic role and regulates p53 and Bcl-2 family proteins expression during ischemia/reperfusion induced apoptosis. Toiber *et al.* [169] showed that overexpression of an *N*-terminally extended synaptic acetylcholinesterase variant, *N*-AChE-S, induced cell death. These newly discover functions of AChE, its interactions with apoptotic proteins, suggest the potential value of AChEIs in AD therapy.

## 5. Conclusions

In this review, recent findings concerning new tacrine hybrids which act as cholinesterase (AChE and BChE), and β-amyloid aggregation inhibitors are summarized. Our attention is focused on dividing these derivatives in following groups: monomeric, homodimeric and heterodimeric congenes, which have the ability to simultaneously bind to both the active and peripheral binding sites of AChE. It is of interest to note the impact of the length and nature of the chain connecting the two skeletons (anchor groups) in relation to the increase in their inhibitory effect. The data suggests that the optimal length of the tether is from 6 to 11 units (methylenes, amides and other). The desolvatation of free energy is also important in this context, suggesting the importance of ligand hydrophobicity for effective interaction with the peripheral side. The most effective inhibitors were found to be the hybrids of tacrine moiety with indanone, 8-hydroxychinoline, indole 3-methoxy-substituted benzene and others. Furthermore, numerous tacrine hybrids have been developed with the aim of improving and enlarging the biological profile beyond its ability to act as ChEIs. Amyloid aggregates are toxic for neurons and influence the direct or indirect origins of the pathological conditions associated with the disease. The precise mechanisms of toxicity have not been fully elucidated; however, there is evidence that the reduction of amyloid deposits leads to the alleviation of the disease's symptoms. Anti-amyloid activity has been observed for several tacrine-based compounds. It was found that both the structure of tacrine derivate and the formation of dimeric or multimeric tacrine conjugates affect the extent of the inhibiting properties of these tacrine derivatives. The development of amyloid aggregation inhibitors would also be able to disaggregate filaments and provide an alternative to existing pharmaceutical strategies. In principle, the inhibitory effect of compounds could take place on different levels, for example, interference with the association of dimmers or oligomers and elongation of filaments. This can be achieved by tight binding of the compounds to the protein monomer and oligomer.

One of the hallmarks of AD is the loss of cholinergic neurons and it is well known that Aβ peptides and higher level of AChE (confirmed in cholinergic neurons in brains affected by AD) are able to induce cell death. The elucidation of the mechanism of neuronal death in brains affected by AD and new functions of AChE are of importance for the development of new drug designs. New types of multifunctional AChEI, with antioxidant potential, an ability to reduce of amyloid aggregation and to interrupt the aberrant influx of Ca^2+^ could be a promising treatment for AD, which explains why hybrid molecules of tacrine are under development as promising drugs.

## Figures and Tables

**Figure 19:**
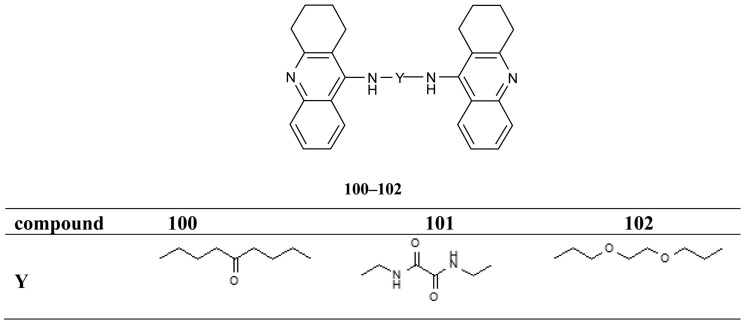
Multifunctional *bis*-tacrines **100–102**.

## Abbreviations

AChE: acetylcholinesterase
ACh: acetylcholine
AD: Alzheimer's disease
Aβ: amyloid beta
ACS: American Chemical Society
*h*AChE: human aceylcholinesterase
*n*AChR: nicotinic acetylcholine receptor
Apaf-1: apoptotic protease-activating factor-1
Bcl-2 family proteins: B-cell lymphoma 2 family proteins
BuChE: butyrylcholinesterase
CAS: catalytic anionic subsite
ChEI: cholinesterase inhibitors
CNS: central nervous system
CTP450: cytochrome P450
CYP2D6: cytochrome P450 2D6
CYP3A4: cytochrome P450 3A4
THA: tetrahydroacridine
5-HT: 5-hydroxytryptamine receptors
7-MEOTA: 9-amino-7-methoxy-1,2,3,4-tetrahydroacridine hydrochloride
nNOS: neuronal nitric oxide synthase
NMDA receptors: *N*-methyl-d-aspartate receptors
PAS: peripheral anionic site
PBT2: tacrine-8-hydroxyquinoline hybrids
